# The Influence of *Piriformospora indica* Colonization on the Root Development and Growth of *Cerasus humilis* Cuttings

**DOI:** 10.3390/plants13111482

**Published:** 2024-05-27

**Authors:** Lu Yin, Pengyan Qu, Dongmei Wang, Songtao Yan, Qinghua Gong, Rui Yang, Yang Hu, Niru Liu, Chunzhen Cheng, Pengfei Wang, Shuai Zhang, Xiaopeng Mu, Jiancheng Zhang

**Affiliations:** College of Horticulture, Shanxi Agricultural University, Jinzhong 030801, China; yl18434764709@163.com (L.Y.); yanyan5235@outlook.com (P.Q.); wdm986@126.com (D.W.); 18634826589@163.com (S.Y.); 18836986625@163.com (Q.G.); yangrui13934147962@163.com (R.Y.); hy16634257765@163.com (Y.H.); liuniru0921@163.com (N.L.); ld0532cheng@sxau.edu.cn (C.C.); 13835436501@163.com (P.W.); wwzs_1990@aliyun.com (S.Z.)

**Keywords:** *Cerasus humilis*, *Piriformospora indica*, plant growth, root development

## Abstract

Numerous studies have shown that the endophytic fungus *Piriformospora indica* has a broad range of promoting effects on root development and plant growth in host plants. However, there are currently no reports on the application of this fungus on *Cerasus humilis*. This study first compared the colonization ability of *P. indica* on 11 *C. humilis* varieties and found that the colonization rate of this fungus on these varieties ranged from 90% to 100%, with the colonization rate of the varieties ‘09-01’ and ‘Nongda 7’ being as high as 100%. Subsequently, the effect of *P. indica* on root development and plant growth of *C. humilis* was investigated using cuttings of ‘09-01’ and ‘Nongda 7’ as materials. *P. indica* colonization was found to increase the biomass of ‘09-01’ and ‘Nongda 7’ plants; root activity, POD enzymes, and chlorophyll content were also significantly increased. In addition, indole-3-acetic acid (IAA) content in the roots of *C. humilis* plants increased after colonization, while jasmonic acid (JA) and 1-aminocyclopropane-1-car- boxylic acid (ACC) content decreased. In conclusion, it has been demonstrated that *P. indica* can promote the growth of *C. humilis* plants by accelerating biomass accumulation, promoting rooting, and enhancing the production of photosynthetic pigments, as well as regulating hormone synthesis.

## 1. Introduction

*Piriformospora indica* is a type of endophytic fungus classified within the *Basidiomycota* phylum, *Hymenomycetes* class, and *Piriformospora* genus. Initially found in the Thar Desert region of northwestern India in 1998, it is named after its pear-shaped thick-walled spores [1].

Comparable with arbuscular mycorrhizal fungi (AMF), which are now widely used in plant production, *P. indica* can form symbiotic relationships with various plants. However, unlike AMF, *P. indica* has a wider host range and can be isolated and cultured in various synthetic media, which facilitates the in-depth studies of its interaction with plants [2]. *P. indica* exhibits a broad spectrum of effects, encompassing not only the stimulation of plant growth and the enhancement of tolerance to both biotic and abiotic stresses but also the induction of systemic resistance in plants [3,4,5]. Therefore, *P. indica* has significant application potential in modern agriculture.

Studies have found that symbiosis with *P. indica* can facilitate plant growth and increase plant yield in different horticultural plants [6]. For example, the symbiotic relationship with *P. indica* leads to notable enhancements in the growth parameters of strawberry plants. These improvements include increased plant height and root proliferation, as well as higher fresh and dry weights. Additionally, there is a significant upregulation observed in strawberry leaf nitrate reductase activity, photosynthetic pigment content, and root vigor [7]. In tomato plants, inoculation with *P. indica* can not only promote the growth of TYLCV-resistant tomato ‘T07-4’ roots but also significantly increase the aboveground biomass and total biomass of susceptible tomato ‘T07-1’; meanwhile, cherry tomatoes inoculated with *P. indica* have significantly enhanced fruit quality, yield, and storage time after harvest [8]. *P. indica*-colonized Brassica napus can accelerate the growth and significantly improves the yield and quality of rapeseed [9].

*Piriformospora indica* can enhance root development by promoting plant hormone secretion. Research has revealed that colonization by *P. indica* can increase POD enzyme activity and IAA content in the *Dimocarpus longan* root system, thereby enhancing the absorption capacity of longan roots for nutrients and promoting the growth of longan cuttings [10]. In rice, colonization by *P. indica* can promote aboveground growth and induce the expression of genes related to growth, increase chlorophyll content, and enhance root vitality. Furthermore, *P. indica* can also induce the secretion of auxin, and these synergistic physiological responses significantly increase the photosynthetic rate of rice, improve the plant’s uptake of mineral nutrients, and thus effectively promote the growth of rice [11].

*Cerasus humilis* is a shrub fruit tree belonging to the Cherry genus, Rosaceae family, and originated in China [12]. It is rich in various nutrients, especially its fruits, which contain abundant organic acids, flavonoids, and unsaturated fatty acids that are beneficial to human health. Due to the high calcium content in its fruits, it is known as the “calcium fruit” among economic fruit trees [13]. *C. humilis* has a wide planting area in China, and, after years of breeding and cultivation, many high-quality *C. humilis* varieties have been screened out by our research team, such as the Nongda Series (Nongda 5, Nongda 6, Nongda 7) and the Jinou Series (Jinou-1, Jinou-2, Jinou-3) [14]. *C. humilis* can be propagated and cultivated in various ways, including dividing, sowing, cutting, grafting, and tissue culture [15]. However, because the planting survival rate of the plant divisions is very low, this type of reproduction is less commonly used in scientific research and practical applications in production [16]. When using sowing propagation, it is not easy to retain the excellent traits of the parent; the survival rate of grafting is more dependent on the suitable rootstock [17]; and tissue culture has a high contamination rate and is prone to massive mortality of the tissue-cultured cuttings, which often occur in the subsequent seedling hardening stages [18]. Therefore, in order to maintain the excellent traits of the parent stock and to improve production efficiency, the propagation of *C. humilis* is usually carried out by cuttings [19]. The quality of *C. humilis* cuttings is directly related to the survival rate after transplantation, so it is crucial to improve the quality of the cuttings [20,21]. Some studies have found that applying plant growth regulators to the foliage can enhance the quality of cuttings [22]. In view of the growth-promoting effect of *P. indica* on other species as mentioned earlier, it can be speculated that it can also promote the growth of *C. humilis* cuttings.

To date, the impact of *P. indica* on the growth and development of *C. humilis* cuttings has not been documented. Therefore, in this study, we colonized *P. indica* during the propagation process of *C. humilis* cuttings and investigated the impact of *P. indica* on the growth and root development of *C. humilis* cuttings by determining the parameters related to growth, root indicators, and photosynthetic pigment content, root activity, and peroxidase content in the roots, as well as the hormone contents of IAA, JA, and ACC in the colonized *C. humilis* cuttings. The results of this study provide scientific support for the seedlings of *C. humilis*, an effective way for its ecological improvement and ecological cultivation in saline and arid areas, and new ideas and practical paths for eco-agriculture and eco-environmental management. However, this study still has some limitations. The current study mainly focused on physiological changes and did not address the molecular level. In the future, transcriptomic and metabolomic studies can be carried out to investigate the molecular mechanism of *P. indica* in promoting the growth of *C. humilis* plants.

## 2. Results

### 2.1. P. indica Colonization Detection Results in Roots of Cerasus humilis Cuttings

**Figure 1:**
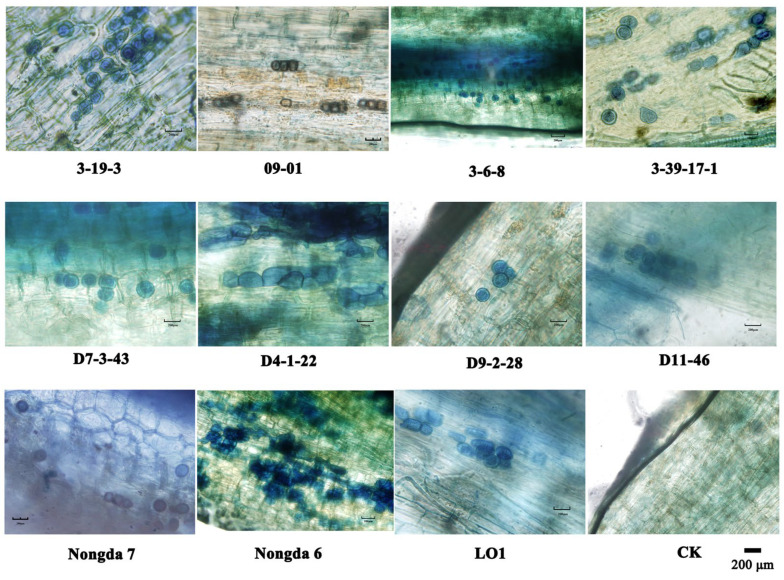
*P. indica* colonization detection results in *Cerasus humilis* roots.

*C. humilis* cuttings co-cultivated with *P. indica* were examined under a microscope after 14 days, as shown in Figure 1. The results showed that *P. indica* successfully colonized the roots of the 11 *C. humilis* varieties mentioned above, indicating that *C. humilis* cuttings can establish a successful symbiotic relationship with *P. indica*.

To study the colonization ability of *P. indica*, the colonization rate of 11 different *C. humilis* varieties was determined. Thirty 1 cm long root segments were randomly selected from each *C. humilis* variety for testing. The results indicated that, among the 11 *C. humilis* varieties tested, the colonization rate of ‘Nongda 7’ and ‘09-01’ reached 100%. The colonization rates of ‘D 4-1-22’ and ‘3-6-8’ were 96%; among the remaining five varieties, the colonization rates of ‘Nongda 6’, ‘LO 1’, ‘D 7-3-43’, ‘D 11-46’, and ‘3-39-17-1’ were all 93%; while the colonization rates of ‘D 9-2-28’ and ‘3-19-3’ were the lowest, at 90%. In summary, *P. indica* was able to successfully colonize most *C. humilis* plants and shows a high affinity.

### 2.2. Influence of P. indica Colonization on the Growth and Development of Cerasus humilis

The growth parameters of the treatment and control groups for ‘09-01’ and ‘Nongda 7’ were measured at 2–5 weeks following inoculation with *P. indica*. Compared with non-colonized *C. humilis* ‘09-01’ cuttings, *P. indica* increased primary root length, number of lateral roots, plant height, and leaf number (Table 1). During the measurement period, successful colonization of *C. humilis* cuttings by *P. indica* resulted in higher fresh and dry weight compared with the untreated group. The growth of ‘09-01’ cuttings was significantly promoted by *P. indica* after 4 weeks of inoculation. The results showed that after 4 and 5 weeks of inoculation, the length of the primary root was notably different from the untreated group (*p* < 0.01), being 1.06-fold and 1.07-fold longer than the control, respectively. At the same time, the number of lateral roots was also notably higher than the control after 4 weeks of inoculation (*p* < 0.05), and the fresh and dry weights were significantly higher than the control (*p* < 0.01). From Table 1, it can be seen that after 3, 4, and 5 weeks of inoculation, the height of the plants colonized by *P. indica* was notably increased compared with the non-inoculated plants (*p* < 0.01), being 1.21-fold, 1.57-fold, and 1.22-fold higher than the untreated plants, respectively. The number of leaves also increased notably (*p* < 0.05), being 1.4-fold, 1.15-fold, and 1.1-fold higher than the untreated, respectively. However, although the stem diameter of the PI group exhibited greater size compared with the CK group, no significant difference was observed.

As can be seen in Table 2, the length of the primary root and the height of ‘Nongda 7’ *C. humilis* cuttings showed significant differences after 4 and 5 weeks of inoculation (*p* < 0.01), being 1.13-fold, 1.17-fold, 1.23-fold, and 1.34-fold of the control, respectively. After 5 weeks of *P. indica* inoculation, the numbers of lateral roots and leaves were markedly higher than those of the untreated group (*p* < 0.05), being 1.34-fold and 1.24-fold higher than the untreated, respectively. *P. indica* did not show any notable change in stem diameter of the ‘Nongda 7’ variety. These growth parameters indicate that *P. indica* has a significant impact on the growth and development of *C. humilis* plants.

**Figure 2:**
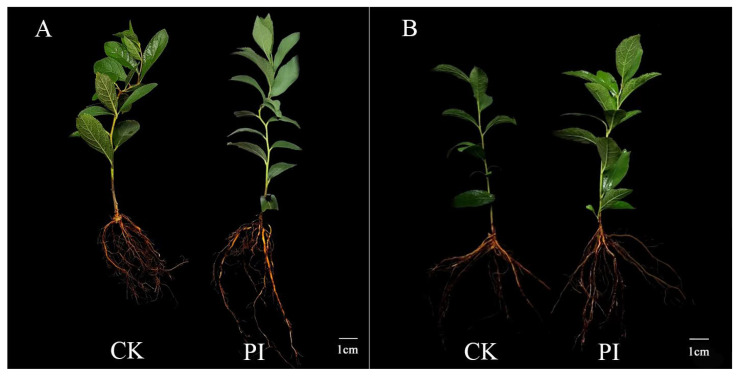
Effect of *P. indica* colonization on the growth and development of *Cerasus humilis* cuttings after 5 weeks. (**A**) is the growth and development map of ‘09-01’; (**B**) is the growth and development map of ‘Nongda 7’; CK: plants without *P. indica* colonization; PI: plants with *P. indica* colonization.

A comparison of the growth status of *P. indica* and *C. humilis* ‘09-01’ and ‘Nongda 7’ after 5 weeks of interaction is shown in Figure 2. The growth status of *C. humilis* cuttings in the performance of the inoculation group was notably superior to that of the control group, with obvious differences in plant height and root length.

### 2.3. Effect of P. indica Colonization on Photosynthetic Pigments in Cerasus humilis Leaves

**Figure 3:**
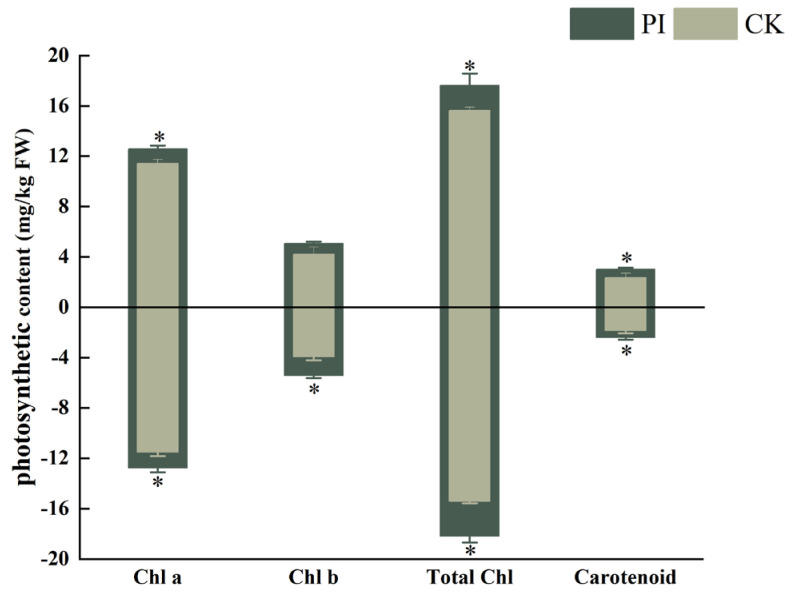
Effect of *P. indica* colonization on photosynthetic pigment content of *Cerasus humilis* cuttings. Horizontal coordinates above represent the ‘09-01’ variety; below represent the ‘Nongda 7’ variety. * indicates significant difference at the 0.05 level (*p* < 0.05).

The chlorophyll content is closely related to plant growth status [23]. The measurement of photosynthetic pigment content can be used to evaluate the growth of plants. Therefore, we measured the photosynthetic pigments in both colonized and non-colonized leaves of *C. humilis* ‘09-01’ and ‘Nongda 7’ plants. Our results revealed a significant increase in chlorophyll a, total chlorophyll, and carotenoid content in the leaves of ‘09-01’ colonized by *P. indica* compared with the non-colonized (*p* < 0.05) as being 1.2-fold, 1.13-fold, and 1.29-fold higher than the untreated group, respectively (Figure 3). Although the chlorophyll a/chlorophyll b ratio in colonized leaves showed a decreasing trend compared with the control, it did not reach statistical significance. Moreover, there was no notable variance in chlorophyll b content between the PI group and the CK group in ‘09-01’.

In ‘Nongda 7’ leaves, *P. indica* colonization resulted in a significant increase in chlorophyll a, chlorophyll b, total chlorophyll, and carotenoid content compared with the control (*p* < 0.05), increasing by 10.9%, 38.1%, 17.8%, and 31.2%, respectively. However, the chlorophyll a/chlorophyll b ratio in colonized ‘Nongda 7’ leaves was significantly lower than the control at only 0.79 times of the control (*p* < 0.05, Figure 3).

### 2.4. Effect of P. indica Colonization on Root Development and Root Activities in Cerasus humilis

One month following successful colonization by *P. indica*, the roots of ‘09-01’ and ‘Nongda 7’ in both the PI and CK groups were scanned in order to measure various parameters, as detailed in Table 3. The ‘09-01’ roots inoculated with *P. indica* showed a significant increase in total length, volume, surface area, projected area, and root tip number compared with the non-colonized group (*p* < 0.01), reaching 1.18, 1.89, 1.80, 1.80, and 1.82 times that of the non-colonized group, respectively. In addition, the average diameter of the roots also increased significantly (*p* < 0.05) by 1.09-fold higher than the control. However, after *P. indica* colonization, only the length, average diameter, and root tip number of ‘Nongda 7’ were significantly increased (*p* < 0.01) by 1.78, 1.85, and 1.55 times that of the control, respectively, while the surface area and projected area were significantly decreased (*p* < 0.05), which were 94.18% and 90.30% of the non-colonized group, respectively. Notably, there was no significant contrast in volume between the PI and CK groups.

**Figure 4:**
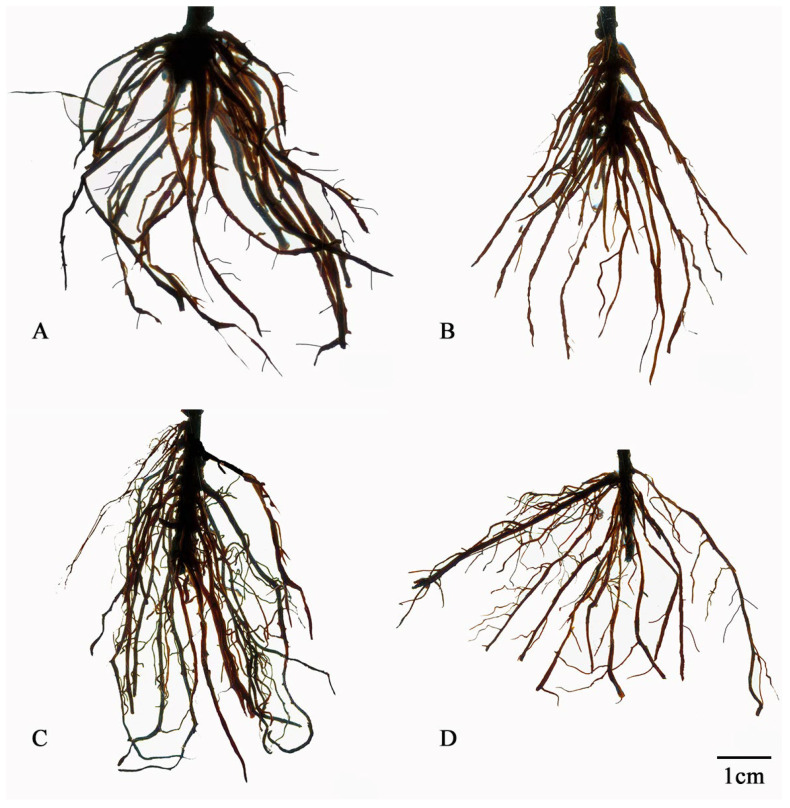
Scanning electron micrographs of root development in *Cerasus humilis* cuttings with and without *P. indica* colonization. (**A**,**B**): ‘09-01’ *Cerasus humilis* root scan of *P. indica* colonization (**A**) and *P. indica* uncolonization (**B**); (**C**,**D**): ‘Nongda 7’ *Cerasus humilis* root scan of *P. indica* colonization (**C**) and *P. indica* uncolonization (**D**).

Figure 4 shows the scanned pictures of *C. humilis* root system. As can be seen from Figure 4, the root system of *C. humilis* inoculated with *P. indica* was significantly better than that of the control group, and there was a significant difference in length and volume between the PI and CK groups.

**Figure 5:**
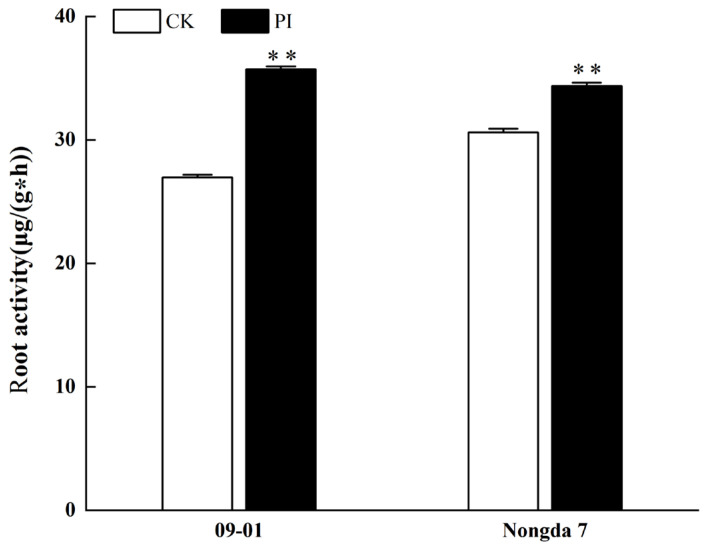
Effect of *P. indica* colonization on root activity of *Cerasus humilis* cuttings. ** indicates significant difference at the 0.01 level (*p* < 0.01).

Root activity is an important indicator for measuring plant health and growth status. This enhancement directly impacts the plant’s ability to absorb water and nutrients, which affects the overall growth of the plant [24]. The influence of *P. indica* colonization on the root vigor of *C. humilis* cuttings was studied, as shown in Figure 5. The results showed that *P. indica* colonization notably enhanced the root activity of both ‘09-01’ and ‘Nongda 7’ (*p* < 0.01), with an increase of 32.5% in ‘09-01’ and 10.9% in ‘Nongda 7’ compared with the control.

### 2.5. Effect of P. indica Colonization on POD and Hormone Levels

**Figure 6:**
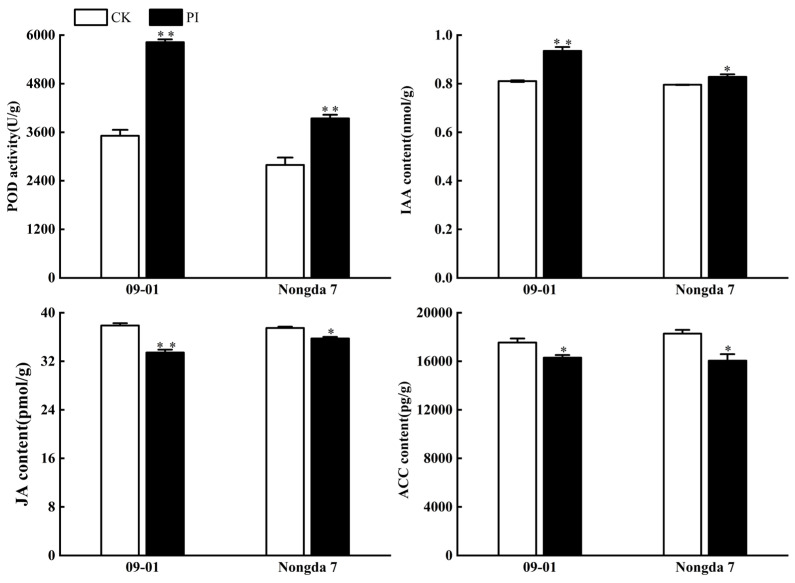
Effect of *P. indica* colonization on POD enzyme activity, IAA, JA, and ACC content of *Cerasus humilis* cuttings. ** indicates significant difference at the 0.01 level (*p* < 0.01); * indicates significant difference at the 0.05 level (*p* < 0.05).

The activity of peroxidase (POD) in roots is closely related to root growth and development [25]. In this experiment, the effect of *P. indica* colonization on the POD enzyme activity in roots of *C. humilis* cuttings was further studied. Compared with the control without *P. indica* inoculation, as depicted in Figure 6, the root POD activity of ‘09-01’ and ‘Nongda 7’ increased significantly (*p* < 0.01), with ‘09-01’ increasing by 65.9% and ‘Nongda 7’ increasing by 41.2%.

To examine *P. indica*’s impact on *C. humilis* plant hormones, the levels of IAA, JA, and ACC in the roots of both the PI and CK groups were measured. The results (Figure 6) indicate that the IAA content of ‘09-01’ roots in the PI group exhibited a notably higher level compared with the untreated group (*p* < 0.01), accounting for 1.15-fold of untreated; the content of ‘Nongda 7’ roots was notably higher than that in the untreated group (*p* < 0.05), which was about 1.04-fold of untreated. Being colonized by *P. indica* notably reduced the JA content in the roots of ‘09-01’ (*p* < 0.01) and ‘Nongda 7’ (*p* < 0.05) to 88.2% and 95.3% of the untreated, respectively. In addition, *P. indica* inoculation significantly reduced the ACC content in the roots of ‘09-01’ and ‘Nongda 7’ (*p* < 0.05), reducing it by 7.6% and 14% compared with the CK, respectively.

## 3. Discussion

Some microorganisms and fungi can form symbiotic relationships with plants, such as rhizobia, which can form nodules with the roots of leguminous plants to convert atmospheric nitrogen into plant-absorbable nitrogen compounds, thus providing important nutrients for plants [26]. In addition, fungi can also form mycorrhizae in symbiosis with plant roots, promoting the growth of both aboveground and underground parts, providing additional moisture and nutrients, and enhancing plant photosynthetic capacity, which is of great significance to agricultural production and the balance of plant ecosystems [27]. Currently, *P. indica* is widely discussed as a beneficial fungus in plants such as strawberry [7], longan [10], and rice [11]. Studies have shown that the growth parameters of barley [28], king grass [29], ryegrass [30], tobacco [31], African chrysanthemum [32], and maize [33] colonized by *P. indica* are higher than those of non-inoculated controls. Additionally, *P. indica* colonization can improve the nutritional quality of black rice [34], promote the germination and growth of Arabidopsis thaliana [35], and increase the aboveground and underground biomass of sweet potato plants [3].

China has abundant germplasm resources of *C. humilis*, which are characterized by early flowering and fruiting, strong resistance, and wide adaptability [36]. This study represents the inaugural demonstration that *P. indica* could successfully colonize the roots of *C. humilis* cuttings, and it was found that the aboveground and underground growth indices of *C. humilis* cuttings colonized by *P. indica* exhibited a significant increase. These findings suggest that *P. indica* possesses the potential to enhance the growth of *C. humilis* cuttings. The results obtained are as follows:

### 3.1. P. indica Establishes Symbiotic Relationship with Cerasus humilis

*Piriformospora indica* is known for its wide range of host plants and can establish symbiotic relationships with various plants. It has been reported that this fungus can colonize more than 200 different plants [6]. Although *P. indica* has been extensively studied on horticultural plants both domestically and internationally, especially on some common plants such as bananas, tomatoes, and cabbage, there have been no reports of the symbiotic relationship between *P. indica* and *C. humilis*. This study found that clear spores could be seen in the rhizosphere of all 11 different *C. humilis* varieties under a microscope, and the infection rate of these varieties exceeds 90%. This finding indicates that *P. indica* can easily colonize the roots of *C. humilis* and that most *C. humilis* varieties have the potential to establish symbiotic relationships with *P. indica*. Additionally, this experiment establishes the foundation for future exploration into the symbiotic interaction between *P. indica* and additional plant species.

### 3.2. P. indica Colonization Promotes the Growth and Root Development of Cerasus humilis Cuttings

Colonization of host plants by *P. indica* can significantly increase the number of plant roots, enhance the root surface area, and promote plant growth. After colonization of Gerbera cuttings by *P. indica*, the root length and aboveground and underground fresh weight of the cuttings are significantly increased; in addition, the numbers of roots and leaves are also significantly increased [32]. *Paeonia lactiflora* infested with *P. indica* had an increase in the fresh mass of its fine roots of 14.14%, 53.05%, and 39.62% at its bud germination, leaf expansion, and bud stage, respectively. The aboveground fresh weight at the leaf expansion and bud stage also significantly increased [37]. After infection by *P. indica*, the size and quality of passion fruit are significantly improved [38]. Previous reports have indicated that colonization of *P. indica* in Tartary buckwheat roots increases the biomass of roots, indicating that *P. indica* colonization promotes the growth of Tartary buckwheat roots [39]. In this study, *P. indica* colonization notably increased the root length, surface area, and plant height of *C. humilis*, thereby promoting the increase in plant biomass. The rapid development of the root system effectively improves the efficiency of nutrient and water absorption, thereby stimulating the growth of the aboveground part of the plant, suggesting that *P. indica* has great potential for application in *C. humilis* seedling cultivation. In addition, from the plant morphology diagram, it can be seen that the plant height of the infected plant ‘09-01’ is significantly higher than that of the treatment group ‘Nongda 7’, indicating that the promoting effect of *P. indica* on different *C. humilis* varieties is different.

### 3.3. P. indica Colonization Greatly Induces the Accumulation of Chlorophyll in Cerasus humilis Leaves

Chlorophyll is a key photosynthetic pigment, and increasing its content helps improve the efficiency of plant photosynthesis, carbon fixation capacity, and stress resistance, thereby promoting plant growth [40]. Numerous studies have indicated that *P. indica* colonization can increase the chlorophyll content of plants such as Anthurium [41], soybeans [42], and *Torreya grandis* cuttings [43]. *P. indica* can enlarge the leaf area of Tartary buckwheat, thereby enhancing its photosynthesis [39]. In the present study, chlorophyll levels were measured in the leaves of both colonized and uncolonized *C. humilis* plants. The results revealed that *P. indica* greatly induced chlorophyll accumulation in the treated group, significantly increasing the content of chlorophyll a, chlorophyll b, and carotenoids, indicating that this fungus can enhance leaf photosynthesis by augmenting photosynthetic pigments.

### 3.4. P. indica Colonization Enhances Root POD Activity Beneficial for Root Growth and Development

The activity level of the POD enzyme is also considered an important indicator for evaluating the growth status of roots and the redox balance in organisms [44]. It not only plays a crucial role in mediating plant stress responses but is also widely used as an indicator for evaluating the growth status of plant roots [45]. During root growth, POD enzymes may influence root morphology and structure by participating in cell division and cell elongation. The changes in enzyme activity reflect the status of the root environment and play a key role in root health and function [46]. It was indicated that the peroxidase in the roots of the tomato plants infected with Verticillium wilt significantly increased after inoculation with *P. indica* [47]. During cadmium stress, *P. indica* notably increased the tobacco’s POD enzyme activity [48]. Similarly, *P. indica* colonization also affected the POD enzyme activity in the roots of passion fruit [49]. It was indicated that the POD enzyme activity was increased in the roots of *C. humilis* cuttings colonized by *P. indica*, suggesting that *P. indica* can increase the POD enzyme activity in the roots of *C. humilis* to promote rooting.

### 3.5. P. indica Promotes Cerasus humilis Root Growth by Inducing IAA Hormone Synthesis, Inhibiting JA and ACC Accumulation

Plant hormones are essential for regulating various aspects of plant growth, including root development [50]. *P. indica* can activate the biosynthesis of auxin and improve the water absorption capacity of roots by increasing the root surface area, which may be a key reason for promoting root growth [51]. Studies have indicated that colonization by *P. indica* stimulates lateral root formation while inhibiting primary root growth in Arabidopsis, which is due to the diffusion of compounds inducing auxin in the co-culture medium of *P. indica* liquid medium and Arabidopsis [52]. At the same time, Arabidopsis can produce indole secondary metabolites to maintain a symbiotic reciprocal relationship with *P. indica* [53]. Studies have reported a notable increase in the IAA in the roots, stems, and leaves of Tartary buckwheat following colonization by *P. indica*. Additionally, the expression level of the ARF2 gene, which is associated with plant growth and fruit development, is significantly enhanced, indicating that *P. indica* can stimulate IAA biosynthesis and increase the upregulation of pertinent genes—processes which facilitate the growth of Tartary buckwheat. The IAA in the roots of two *C. humilis* varieties measured in this experimental was notably higher than that of the untreated group, suggesting that *P. indica* can activate IAA synthesis and promote root development in *C. humilis*, consistent with the above conclusions.

JA plays a key role in plant root growth, mainly inhibiting root growth by limiting cell division in the meristematic tissue zone and cell elongation in the elongation zone. It has been shown that JA negatively regulates root growth under B-deficient conditions by activating the JA signaling pathway through *JAR1* [54,55]. Fortunately, interaction of *P. indica* with the plant root system reduces jasmonic acid levels in the plant, which in turn promotes the growth and development of plant roots [56]. When *P. indica* colonizes the roots of longan, the content of JA in the roots is significantly inhibited [10]. ACC is a precursor of ethylene synthesis, and ethylene is an important plant hormone involved in many growth and development processes [57]. In plants, the interaction of jasmonic acid (JA) with ethylene can be manifested by the formation of the JA–ACC chemical complex [55]. It has been found in studies that high concentrations of the JA–ACC chemical complex inhibited root growth of the *jar1-1* mutant in Arabidopsis, indicating that high concentrations of ethylene inhibit root growth [58,59]. In this experimental study, it was indicated that colonization of *P. indica* notably inhibits the level of JA and ACC in the roots of *C. humilis*; therefore, we speculated that the promotion of root development by *P. indica* inoculation is related to the decrease in these two hormones.

## 4. Materials and Methods

### 4.1. Plant Materials and Fungal Preparation

In this study, 11 *C. humilis* varieties (‘Nongda 7’, ‘Nongda 6’, ‘LO1’, ‘D 9-2-28’, ‘D 7-3-43’, ‘D 4-1-22’, ‘D 11-46’, ‘3-19-3’, ‘09-01’, ‘3-6-8’, ‘3-39-17-1’) were used as experimental materials, and subsequent experiments were carried out using ‘09-01’ and ‘Nongda 7’ as indicator measurement materials.

The *Piriformospora indica* strain was preserved by the *C. humilis* team at Shanxi Agricultural University. The *P. indica* spore suspension was prepared following the procedure method by Cheng et al. [60] and subsequently adjusted to the final concentration of 2 × 10^7^ spores/mL using a hemocytometer counting plates for subsequent experiments.

Healthy semi-lignified branches with a length of 8–10 cm were collected and planted in tray cells, ensuring that each cutting had 4 intact leaves. After normal management for one month, the *C. humilis* cuttings of the above-mentioned 11 varieties with good health, consistent growth, and free from pests and diseases were divided into two groups. One group had the young roots of *C. humilis* soaked in the fermentation solution for 6 h (PI group), and the other group had an equal volume of PDB liquid medium as the control (CK group). The cuttings were placed in a small plastic greenhouse for subsequent management.

### 4.2. Piriformospora indica Colonization Detection

Taipan blue staining observation: After co-cultivating with *P. indica* for 2 weeks, 5 PI cuttings were randomly selected. After rinsing the roots of *C. humilis* with tap water, they were cut into 1 cm long segments and mixed. They were treated with 5% KOH at 90 °C for 1 h, followed by 2% HCl at indoor temperature for 5 min. After discarding the solution, the samples were treated with 0.05% Taipan blue reagent at 90 °C for 60 min. The solution was then discarded, and the samples were subjected to decolorization at room temperature for 2 days. Six segments were selected from each seedling, for a total of 30 segments, and the colonization of root spores was observed under a microscope. The successfully colonized ‘09-01’ and ‘Nongda 7’ cuttings were used for subsequent experiments. Infestation rate (%) = (number of infested root segments/total number of root segments) × 100%.

### 4.3. Determination of Plant Growth Parameters

At 2, 3, 4, and 5 weeks after inoculation with *P. indica*, both colonized and non-colonized ‘09-01’ and ‘Nongda 7’ cuttings were collected, rinsed, and used for the measurement of plant growth parameters, including plant height (cm), primary root length (cm), stem thickness (cm), number of lateral roots, leaf number, aboveground fresh, root fresh, total fresh weight (g), aboveground dry, root dry, and total dry weight (g). The aboveground and underground dry weights were measured after having been dried at 55 °C until constant weight. Five cuttings were selected for each parameter.

### 4.4. Determination of Photosynthetic Pigments in Cerasus humilis Leaves

After one month of successful colonization of ‘09-01’ and ‘Nongda 7’ cuttings by *P. indica*, the chlorophyll content of *C. humilis* leaves was measured using ethanol extraction [61]. Five cuttings were randomly selected from each group, and the experiment was repeated 3 times. Chlorophyll a, chlorophyll b, and carotenoids were determined by measuring absorbance at wavelengths of 665 nm, 649 nm, and 470 nm, respectively, using the following formula:

C_a_ = 13.95 × A_665_ − 6.88 × A_649_; C_b_ = 24.96 × A_649_ − 7.32 × A_665_; C_x.c_ = (1000 × A_470_ − 2.05 × C_a_ − 114.8 × C_b_)/245; Total chlorophyll = C_a_ + C_b_

### 4.5. Determination of Root Development and Activities in Cerasus humilis

The root development and root activity of *C. humilis* were determined one month after successful colonization by *P. indica*. The roots of the ‘09-01’ and the ‘Nongda 7’ PI group and CK group were scanned using the Root126 phenotype analysis system. The measured parameters included length (m), average diameter (mm), volume (mm^3^), surface area (mm^2^), projected area (mm^2^), and number of root tips. 

According to the “Experimental Guide of Plant Physiology” [61], the root activity of colonized and non-colonized *C. humilis* plants by *P. indica* was determined using the triphenyltetrazolium chloride (TTC) method: weigh 0.5 g of *C. humilis* seedling root tip samples; add 5 mL of 0.4% TTC solution and 5 mL of phosphate buffer solution; immerse the solution in the roots; and incubate in a constant temperature incubator at 37 °C for 3 h in the dark. After 3 h, add 2 mL of 1 mol/L sulfuric acid to stop the reaction. Remove and dry the roots; grind them with ethyl acetate to extract methylazide; wash the residue with a small amount of ethyl acetate and transfer it to a test tube; and finally, make up to 10 mL with ethyl acetate. The reduction amount of tetrazolium was measured at a wavelength of 485 nm. Three biological replicates were performed for each parameter.

### 4.6. Determination of Root Antioxidant Enzyme Activities and Hormone Levels in Cerasus humilis

After co-culturing with *P. indica* for one month, the activity of root peroxidase (POD) in colonized and non-colonized ‘09-01’ and ‘Nongda 7’ *C. humilis* roots was determined using the guaiacol method [61]. Three biological replicates were performed for both the control and treatment groups.

Enzyme preparation: place 0.1 g of PI group and CK group *C. humilis* seedling roots in a pre-cooled mortar and add 2 mL of 50 mM pH 7.8 phosphate buffer solution on ice. Grind the solution and pour it into a 10 mL centrifuge tube. Wash the mortar twice with 2 mL of extraction solution and combine the 6 mL of solution in the centrifuge tube. After shaking and letting stand for 2 min, transfer the solution to a 5 mL centrifuge tube (take 4 mL of liquid) and then centrifuge at 10,000 rpm for 15 min and store in a 4 °C refrigerator for later use.

POD activity determination method: add 200 μL of enzyme solution to a colorimetric dish and then add 3 mL of reaction solution and immediately start the timer. Take the initial absorbance measurement at a wavelength of 470 nm and note the final absorbance after 3 min. POD activity = [(△A × v)/w × v_t_ × t] × n.

△A—change in absorbance within 3 min; v—total volume of enzyme extraction solution; v_t_—volume of enzyme solution used for measurement; w—sample fresh weight; t—reaction time; n—dilution factor (if the enzyme activity is too high, the enzyme solution needs to be diluted). 

To determine the hormone content of colonized and uncolonized *C. humilis* roots by *P. indica*, take 0.1 g of root sample and add 9 times the volume of phosphate buffer solution for thorough homogenization. After completion, centrifuge at 2500 rpm for 20 min to collect the supernatant. The content of IAA, JA, and ACC in the roots of ‘09-01’ and ‘Nongda 7’ *C. humilis* was determined using the Plant140 enzyme-linked immunosorbent assay kit (Solarbio, Beijing, China) and the Multiskan SkyHigh 500C microplate reader (Thermo Fisher Scientific, Shanghai, China). Three replicates were performed for each parameter.

### 4.7. Statistics Analysis

The outcomes of the aforementioned parameters were presented as the mean standard deviation (SD) from a minimum of three replicates. Statistical analysis was conducted utilizing SPSS 15.0 software. When there were only 2 groups for comparison, an independent variable *t*-test was used for significance analysis (*p* < 0.05). The images were plotted using Origin 2021 software.

## 5. Conclusions

The results showed that *P. indica* can easily colonize the roots of *C. humilis*, and fungal colonization increases the photosynthetic pigment content in the leaves of *C. humilis* cuttings, thereby promoting the photosynthesis and growth of *C. humilis* cuttings. Additionally, *P. indica* enhances the absorption capacity of nutrients in *C. humilis* plants by stimulating the synthesis of IAA, increasing the activity of POD enzymes and root vitality, and inhibiting the content of JA and ACC, thus promoting the growth of *C. humilis* plants.

## Figures and Tables

**Table 1:** Effect of *P. indica* on agronomic traits of ‘09-01’ in different periods. ** indicates significant difference at the 0.01 level (*p* < 0.01); * indicates significant difference at the 0.05 level (*p* < 0.05).

	2W		3W		4W		5W	
	CK	PI	CK	PI	CK	PI	CK	PI
Rootlet length (cm)	4.09 ± 0.10	6.49 ± 0.05	6.12 ± 0.12	7.08 ± 0.11	7.79 ± 0.10	8.28 ± 0.09 **	9.40 ± 0.10	10.03 ± 0.21 **
Lateral root number	3.00 ± 0.58	4.00 ± 1.00	5.00 ± 0.96	7.00 ± 0.82	7.00 ± 1.50	10.25 ± 0.96 *	10.67 ± 1.15	12.33 ± 0.58 *
Plant height (cm)	5.66 ± 0.66	6.63 ± 0.55	7.36 ± 0.32	8.88 ± 0.44 **	8.32 ± 0.16	13.03 ± 0.49 **	16.57 ± 1.06	20.20 ± 0.69 **
Stem thickness	1.13 ± 0.14	1.12 ± 0.15	1.44 ± 0.12	1.61 ± 0.04	1.56 ± 0.06	1.78 ± 0.04	1.95 ± 0.04	2.12 ± 0.01
Blade number	4.67 ± 0.58	4.33 ± 0.58	6.25 ± 0.50	8.75 ± 0.96 *	12.00 ± 0.82	13.75 ± 0.96 *	16.00 ± 0.66	17.67 ± 0.58 *
Aboveground part fresh	0.29 ± 0.03	0.50 ± 0.04	0.39 ± 0.04	0.53 ± 0.05	0.42 ± 0.03	0.84 ± 0.04 **	1.15 ± 0.06	1.33 ± 0.03 **
Root fresh weight (g)	0.16 ± 0.02	0.42 ± 0.03	0.31 ± 0.03	0.45 ± 0.03	0.34 ± 0.04	0.60 ± 0.02 **	0.53 ± 0.03	1.23 ± 0.03 **
Plant fresh weight (g)	0.45 ± 0.05	0.94 ± 0.05	0.70 ± 0.07	0.98 ± 0.06	0.76 ± 0.07	1.44 ± 0.05 **	1.68 ± 0.08	2.56 ± 0.05 **
Aboveground part dry	0.14 ± 0.03	0.18 ± 0.03	0.16 ± 0.05	0.27 ± 0.04	0.19 ± 0.02	0.35 ± 0.02 **	0.39 ± 0.04	0.59 ± 0.04 **
Root dry weight (g)	0.10 ± 0.02	0.13 ± 0.03	0.12 ± 0.03	0.17 ± 0.04	0.16 ± 0.02	0.25 ± 0.03 **	0.26 ± 0.05	0.40 ± 0.04 **
Plant dry weight (g)	0.24 ± 0.05	0.31 ± 0.06	0.27 ± 0.05	0.44 ± 0.04	0.35 ± 0.03	0.60 ± 0.06 **	0.64 ± 0.02	0.98 ± 0.04 **

**Table 2:** Effect of *P. indica* on agronomic traits of ‘Nongda 7’ in different periods. ** indicates significant difference at the 0.01 level (*p* < 0.01); * indicates significant difference at the 0.05 level (*p* < 0.05).

	2W		3W		4W		5W	
	CK	PI	CK	PI	CK	PI	CK	PI
Rootlet length (cm)	7.41 ± 0.10	7.59 ± 0.26	8.18 ± 0.14	9.82 ± 0.10 *	9.07 ± 0.15	10.26 ± 0.28 **	9.27 ± 0.32	10.93 ± 0.15 **
Lateral root number	3.67 ± 0.58	5.00 ± 1.00	4.33 ± 0.58	5.67 ± 0.95	5.00 ± 1.00	6.00 ± 1.00	5.67 ± 0.58	7.00 ± 0.58 *
Plant height (cm)	7.43 ± 0.41	8.13 ± 0.44	8.35 ± 0.19	9.43 ± 0.55	10.76 ± 0.46	14.37 ± 0.12 **	13.63 ± 0.32	16.77 ± 0.50 **
Stem thickness	1.58 ± 0.04	1.47 ± 0.08	1.62 ± 0.03	1.64 ± 0.03	1.67 ± 0.05	1.68 ± 0.05	1.68 ± 0.03	1.70 ± 0.02
Blade number	5.55 ± 0.47	5.67 ± 0.58	6.33 ± 0.58	6.67 ± 0.58	10.33 ± 0.65	12.67 ± 0.58	11.33 ± 0.96	14.00 ± 0.85 *
Aboveground part fresh	0.33 ± 0.02	0.45 ± 0.03	0.41 ± 0.03	0.50 ± 0.02	0.53 ± 0.04	0.73 ± 0.05 *	0.56 ± 0.05	0.81 ± 0.03 *
Root fresh weight (g)	0.30 ± 0.01	0.36 ± 0.02	0.32 ± 0.02	0.37 ± 0.02	0.30 ± 0.03	0.33 ± 0.01	0.40 ± 0.03	0.39 ± 0.03
Plant fresh weight (g)	0.63 ± 0.04	0.81 ± 0.04	0.73 ± 0.01	0.87 ± 0.01	0.83 ± 0.05	1.06 ± 0.03 *	0.96 ± 0.06	1.20 ± 0.04 *
Aboveground part dry	0.18 ± 0.03	0.21 ± 0.02	0.23 ± 0.03	0.26 ± 0.03	0.26 ± 0.02	0.35 ± 0.05	0.28 ± 0.04	0.47 ± 0.03 **
Root dry weight (g)	0.12 ± 0.01	0.13 ± 0.02	0.17 ± 0.02	0.18 ± 0.02	0.16 ± 0.01	0.19 ± 0.01	0.22 ± 0.03	0.24 ± 0.02 **
Plant dry weight (g)	0.30 ± 0.03	0.34 ± 0.04	0.40 ± 0.05	0.45 ± 0.03	0.42 ± 0.02	0.55 ± 0.09	0.50 ± 0.02	0.70 ± 0.04 **

**Table 3:** Effects of inoculation on root parameters of *Cerasus humilis* with and without *P. indica* colonization. ** indicates significant difference at the 0.01 level (*p* < 0.01); * indicates significant difference at the 0.05 level (*p* < 0.05).

	09-01		Nongda 7	
	CK	PI	CK	PI
Length (m)	0.80 ± 0.01	0.94 ± 0.03 **	0.74 ± 0.02	1.32 ± 0.02 **
Average diameter (mm)	0.86 ± 0.03	0.94 ± 0.05 *	0.57 ± 0.06	1.06 ± 0.04 **
Volume (mm^3^)	551.36 ± 13.18	1043.56 ± 17.91 **	924.50 ± 28.77	870.68 ± 32.78
Surface area (mm^2^)	1440.02 ± 23.73	2594.65 ± 37.31 **	2204.5 ± 81.58	1990.56 ± 91.23 *
Projected area (mm^2^)	458.37 ± 7.56	825.90 ± 9.40 **	791.16 ± 7.54	612.39 ± 5.53 *
Number of root tips	61.33 ± 4.04	111.67 ± 7.02 **	93.00 ± 2.65	144.00 ± 2.00 **

## Data Availability

Data are contained within the article.
